# The vertebrate middle and inner ear: A short overview

**DOI:** 10.1002/jmor.20880

**Published:** 2018-08-17

**Authors:** Cathrin Pfaff, Julia A. Schultz, Rico Schellhorn

**Affiliations:** ^1^ University of Vienna, Department of Palaeontology Vienna Austria; ^2^ University of Chicago, Department of Organismal Biology and Anatomy Chicago Illinois USA; ^3^ Rheinische Friedrich‐Wilhelms‐Universität Bonn, Steinmann Institut für Geologie, Mineralogie und Paläontologie Bonn Germany

**Keywords:** auditory ossicles, bony labyrinth, otic region, vestibular system

## Abstract

The evolution of the various hearing adaptations is connected to major structural changes in nearly all groups of vertebrates. Besides hearing, the detection of acceleration and orientation in space are key functions of this mechanosensory system.

The symposium “show me your ear – the inner and middle ear in vertebrates” held at the 11th International Congress of Vertebrate Morphology (ICVM) 2016 in Washington, DC (USA) intended to present current research addressing adaptation and evolution of the vertebrate otic region, auditory ossicles, vestibular system, and hearing physiology. The symposium aimed at an audience with interest in hearing research focusing on morphological, functional, and comparative studies. The presented talks and posters lead to the contributions of this virtual issue highlighting recent advances in the vertebrate balance and hearing system. This article serves as an introduction to the virtual issue contributions and intends to give a short overview of research papers focusing on vertebrate labyrinth and middle ear related structures in past and recent years.

## 1 INTRODUCTION

The vertebrate ear region, ‐ middle ear structures and labyrinth ‐ is one of the most intricate anatomical systems and has been in the focus of various studies over the centuries. With the increasing application of high resolution and noninvasive computer tomography since the 1980s, 3D visualization and their respective analyses became essential for anatomical studies of the middle and inner ear.

The vertebrate ear combines two main functions: balance and hearing. The vestibular part of the inner ear is the organ of equilibrium detecting head and body movements during locomotion (e.g., swimming, running, and flying). The semicircular canals (SCs) (three in gnathostomes: anterior semicircular canal, posterior semicircular canal, lateral/horizontal semicircular canal) of the vestibular system detect angular acceleration, while utricule and saccule detect linear acceleration (e.g., Breuer, 1903; de Burlet, 1934; Retzius, 1881). Sound conduction and perception in living vertebrates is performed by the stapedial structure and lagenar recess (in nonmammalian tetrapods), or middle ear ossicles and the cochlea (in mammals; e.g., Doran, 1878; Fleischer, 1978). Those ossicles transmit vibrations from the eardrum (except in nonmammalian tetrapods; Clack & Anderson, 2016) to the fenestra vestibuli/fenestra ovalis of the vestibule (e.g., Fleischer, 1973; Gray, 1907).

This contribution provides a short overview of the history of research related to the vertebrate middle and inner ear, without any claim on completeness. Furthermore, it highlights evolutionary concepts and serves as an introduction to the virtual issue of the Journal of Morphology uniting research papers related to function and morphology of auditory ossicles of the middle ear and membranous as well as bony labyrinth (skeletal labyrinth: prismatic and globular calcified cartilage in chondrichthyan fishes [Mason & Summers, 2006]) in vertebrates, motivated by the symposium. This virtual issue of the Journal of Morphology provides a unique opportunity to collectively present the current state of research as a synopsis of the symposium.

### 1.1 Middle ear

The evolutionary origin of the vertebrate middle ear related structures is considered to lie in the hyostyl jaw apparatus (composed of mandibular arch, hyomandibular, and ceratohyal bones) of elasmobranchs (Gegenbaur 1872, 1898, Goodrich, 1930, reviewed by Gaupp, 1898). However, form, function, and homology of these middle ear elements (auditory ossicles) of vertebrates have been contradictory discussed since the beginning of the 16th century (reviewed by Gitter, 1990).

Étienne Geoffroy Saint‐Hilaire (1818) and Lorenz Oken (1825) were among the first who postulated that the mammalian middle ear ossicles are homologous to the opercular bones of teleosts (contradictory views: Rudolphi, 1821; Huschke, 1824; Henson, 1974). But hearing in cartilaginous and bony fishes is different from hearing in tetrapods and is convergently realized by a great variety of anatomical settings (Fay & Popper, 1978; Popper & Fay, 1977; Retzius, 1881; Tavolga, Popper, & Fay, 1981; Wever, 1974).

In sharks, hearing is realized by the two displacement systems (cupulae with sensory hair cells) of the lateral line receptors and the labyrinth (Dijkgraaf, 1963; Harris & van Bergeijk, 1962), whereas in bony fishes several sound transmission systems exist. In the latter, hearing is realized by the close relationship of the swim bladder and ear region (e.g., notopterids, beryciforms) or by small gas‐filled vesicles extending from the swim bladder (e.g., clupeids, mormyrids; Platt & Popper, 1981). The size of these vesicles (Alexander, 1959; Blaxter, 1981; Poggendorf, 1952) and the distance to the inner ear influence the hearing ability of the specimens (Coombs & Popper, 1979; Jerkø, Turunen‐Rise, Enger, & Sand, 1989). The swim bladder itself plays an important role in the hearing process of fishes like the detection of long‐distance frequencies and spatial hearing. In comparison, fishes without an air‐filled cavity are not able to hear sounds outside their near‐field (Schellart & Popper, 1991; Siler, 1969). A well investigated system for sound transmissions are the Weberian ossicles (tripus, intercalarium, scaphium, and claustrum) of extant and extinct otophysine fishes. A double chain of ossicles connects the inner ear labyrinth with the swim bladder to enhance hearing sensitivity (see Friedman & Giles, 2016). An analogous structure to this mechanic sensory system is also found in Chanidae (Actinopterygii, Gonorynchiformes), which presumably enhances audition (Rosen & Greenwood, 1970). Absolute sensitivity and hearing bandwidth depends on the anatomical relationships of the hearing related structures, but more importantly on the type of connection between the otolith organs and gas‐filled spaces (Blaxter, 1981).

Hearing abilities are strongly depending on the acoustic environment (Hawkins, 1981). When the earliest vertebrates left the water to conquer terrestrial habitats, they were still primarily aquatic animals (Clack, 1997, 2002; Coates & Clack, 1991), but were suddenly confronted with airborne sound. The otic region of the earliest tetrapods from the Late Devonian (*Acanthostega gunnii*, *Ichthyostega* spp.) differs anatomically from the derived amniote ear (e.g., in mammals) in showing large and bulky stapedial bones (relative to braincase size) capable of the detection of substrate‐borne vibrations (Clack et al., 2003; Clack & Anderson, 2016). They possibly could only hear low‐frequency, ground‐ or water‐borne sounds, which are concordant with the supporting‐brace function of the stapes connecting the cranium (Manley, 2010).

One of the key innovations of extant sauropsids, mammals, and anurans (except of gymnophiones and caudates) is the tympanum (tympanic membrane) overlying an air‐filled middle ear cavity with ossicles transmitting the sound to the oval window of the inner ear (Hetherington, 1992). This type of middle ear for transmitting airborne sound evolved multiple times independently within terrestrial tetrapods, reflected for example by the position of the tympanum in relation to Meckel's cartilage or the existence of the extrastapes in sauropsids (Gaupp, 1913; Lombard & Bolt, 1979), but also found in ontogenetic sequences of developmental studies (Tucker, Watson, Lettice, Yamada, & Hill, 2004; reviewed by Tucker, 2017).

In anuran amphibians, the stapes runs through connective and muscle tissue to reach the oval window (Jaslow, Hetherington, & Lombard, 1986; Wever, 1985). At its distal end it connects to an extrastapes (Mason & Narins, 2002) that passes medially (Hetherington, 1992). Covered by a tympanum, the convergently evolved opercularis system is found in metamorphous anurans and salamanders (Hetherington, 1992; Hetherington, Jaslow, & Lombard, 1986; Kingsbury & Reed, 1909; Wever, 1985), which does not occur in completely aquatic and neotenic salamanders and gymnophiones (Hetherington, 1992). This system consists of an opercularis muscle originating from the pectoral girdle and inserting on the otic operculum, which rests in the oval window (Hetherington, 1992). Its function is controversially discussed (Eiselt, 1941; Hetherington, 1985, 1992; Kingsbury & Reed, 1909; Lombard & Straughan, 1974; Wever, 1979, 1985), but it has commonly been assumed that the opercularis system represents the reduction and loss of the middle ear covered by a tympanic membrane possibly due to the lack of vocalization of the respective species (Hetherington, 1992; contradictory view: McDiarmid, 1971, Trueb & Alberch, 1985, Jaslow et al., 1986) and is also considered as a nontympanic pathway of sound perception. However, auditory systems for transmitting sound are highly influenced by evolutionary changes of the body size and shape of amphibians and as a result those changes determined the evolution of the mechanisms of the acoustic reception (Hetherington, 1992).

Some sauropsids (e.g., *Ophisaurus apodus, Gecko gecko, Eublepharis maculatus)* are able to close their external acoustic meatus with a meatal muscle, possibly for protection against mechanical damage or their own sound (Wever, 1978). In sauropsids, the tympanic middle ear consists of the tympanic membrane connecting to the proximal element of the columella. The footplate of the columella sits in the oval window and transmits the detected sound waves to the scala vestibuli of the membranous labyrith (Wever, 1978). In some nonmammalian amniotes an extracolumella can occur between tympanic membrane and columella (e.g., in archosaurs and turtles). Whereas the columella is an ossified slender rod with an expanded footplate articulating into the oval window, the extracolumella is entirely cartilaginous showing a number of processes for stiffening the tympanic membrane and transmitting the vibrations. The joint between these two middle ear elements are flexible but can also be a solid union strengthened by a layer of connective tissue (Wever, 1978).

The transition from early to advanced nonmammalian amniotes represents one of the major key steps in the evolution of the vertebrate middle ear. Via the “single‐ossicle” middle ear (stapes) of nonmammalian amniotes, sounds bouncing off the skin surface are transmitted to the cochlear duct (Manley, 2010).

In the 19th century, Georges Cuvier (1805) described the middle ear bones of amphibians, sauropsids (lepidosaurs, archosaurs), and mammals, in detail without comparing their anatomical differences and evolutionary history. Following these first anatomical descriptions of the middle ear ossicles, Carus (1818) and Reichert (1837) published the theory that the mammalian stapes is a homologue of the columella auris of nonmammalian amniotes (reviewed by Russell, 1916). Reichert (1837) hypothesized that two of the mammalian middle ear ossicles (malleus, incus) derived from upper (palatoquadrate) and lower jaw elements (Meckel's cartilage) of primitive gnathostomes. This was followed by a series of scientifically important descriptions of the nonmammalian amniote ear (e.g., Hasse, 1871; Kuhn, 1882; Retzius, 1880, 1884). It took additional 75 years to modify and validate Reichert's theory (Gaupp, 1911a, 1911b, 1913). The ‘Reichert‐Gauppsche Theorie’ (Gaupp, 1911a; Reichert, 1837) basically describes the revolutionary view that the primary jaw joint between quadrate and the articular lost its function in mammals, and that a secondary jaw joint situated between the squamosal and dentary bone is developed. During this process, dentary elements detach from the lower jaw and transform into delicate bony elements for sound conduction that are integrated into the middle ear cavity inside the mammalian skull (Anthwal, Joshi, & Tucker, 2013; Takechi & Kuratani, 2010). These works fundamentally contributed to the finding that the stapes of mammals is homologous to the columella of nonmammalian amniotes and the origin of this bone is considered to be the second pharyngeal arch of ancestral gnathostomes. These results from comparative analyses were recently confirmed by genetic labeling experiments (Thompson, Ohazama, Sharpe, & Tucker, 2012).

The middle ear of modern mammals composed of malleus (hammer), incus (anvil), and stapes (stirrup) is laterally restricted by the ectotympanic ring and completely detached from the mandible (Allin, 1975; Allin & Hopson, 1992). Complex bone reorganization of the mandible occurred during detachment of the mammalian middle ear, which is well supported by fossil evidence (e.g., Allin & Hopson, 1992; Kermack, Mussett, & Rigney, 1981; Luo, Schultz, & Ekdale, 2016; Manley & Sienknecht, 2013; Martin & Luo, 2005; Meng, Bi, Zheng, & Wang, 2016; Meng, Wang, & Li, 2011). Clearing and staining techniques using ontogenetic sequences drew an even clearer picture of the evolution of the mammalian middle ear in more recent years (Anthwal et al., 2013; Ramírez‐Chaves et al., 2016a; Ramírez‐Chaves, Weisbecker, Wroe, & Phillips, 2016b; Rich, Hopson, Musser, Flannery, & Vickers‐Rick, 2005; Urban et al., 2017). Developmental genetics and gene patterning of early ontogenetic stages of extant mammalian species helped to identify complex homoplasies (convergences and reversals) in the evolution of the mammalian secondary jaw joint and middle ear (Maier, 1990; Zeller, 1993; Tucker et al., 2004; Luo, 2011; Kitazawa et al., 2015). The functional shift of elements used for feeding into elements used for hearing is impressively traceable in advanced cynodonts (e.g., *Diarthrognathus*), which possess both functional jaw joints (primary and secondary) at the same time (Allin, 1975; Hopson, 1966; Takechi & Kuratani, 2010). The segregation of the middle ear ossicles from the lower jaw and incorporation into the basicranium is also traceable in the ontogeny of marsupials (Maier, 1990). Even if this morphological transition is well understood, the evolutionary chronology is still controversially discussed (e.g., Segall, 1969, 1973). Doran (1878) conducted the most comprehensive anatomical study of the mammalian middle ear ossicles focusing on the evolutionary morphology of auditory ossicles. Special emphasis must be placed on the work of Fleischer (1973) who distinguished distinct types of articulation between malleus and incus (e.g., freely mobile, micro) and discussed the implications for the mammalian phylogeny and evolution. However, both Doran (1878) and Fleischer (1973) made mistakes in the anatomy of the Processus anterior mallei and ignored its additional processus internus articularis (Maier & Ruf, 2016).

### 1.2 Inner ear

The inner ear (labyrinth) of vertebrates comprises two main functions: (a) balancing of the individual using the ampullae of the three SCs and the sensory field of utriculus and sacculus, and (b) hearing ability with the sensory hair cells of basilar papilla of the lagena of nonmammalian amniotes (the homologous structure in mammals is the coiled cochlea with the organ of Corti). The inner ear is composed of a membranous system (membranous labyrinth) filled with endolymphatic fluid enclosed inside a bony endocast (bony labyrinth), filled with perilymphatic fluid. Both fluids differ in their concentration of potassium and sodium (e.g., Ferrary, Huy, Roinel, Bernard, & Amiel, 1988; Sterkers, Ferrary, & Amiel, 1988; Thalmann & Thalmann, 1999). The membranous system of the inner ear is separated in pars superior (utricule and three semicircular canals) and pars inferior (saccule and cochlear duct; de Burlet, 1929).

In all vertebrates, movement of the animal head and body causes movement of the endolymphatic fluid. Physiologically, this initiates mechanical displacement of the hair cells of the sensory fields of the ampullae and maculae in the inner ear and causes nerve stimulation. The ampullae of the SCs filled with fluid detect rotational movement of the organism, whereas the otolithic layer of the maculae of the vestibule detects longitudinal and vertical acceleration during locomotion (e.g., Ekdale, 2013; Steinhausen, 1933; Van Egmond, Groen, & Jongkees, 1949).

In jawless vertebrates, the number of SCs varies (Retzius, 1881; Stensiö, 1927). The vestibular system of hagfishes shows one semicircular canal (Jørgensen, Shichiri, & Geneser, 1998), while lampreys possess two symmetric horizontal ducts in each labyrinth (Maklad, Reed, Johnson, & Fritzsch, 2014). In the teleost ear, the highest anatomical variation is seen in the lagena and the sacculus, whereas the semicircular canals and the utriculus are less variable (Platt & Popper, 1981). In terrestrial vertebrates, the middle ear communicates with the inner ear via the oval window (fenestra vestibuli) by moving the footplate of the stapes. The oval window represents a remnant of the lateral otic fissure in tetrapodomorph fishes (e.g., *Eusthenopteron*) and early tetrapods, which remains unossified during embryogenesis (Clack, 2016). The round window (fenestra cochleae) is covered by the secondary tympanic membrane (Zeller, 1985), which equalizes pressure induced by the movement of the lymphatic liquid within the membranous ducts of the inner ear (de Burlet, 1934).

In the 16th century, Bartholomeus Eustachius discovered the cochlear part of the ear, which was denoted by Gabriel Falloppio as “cochlea” in 1561 (Gitter, 1990). Politzer (1907) and more recently Hachmeister (2003) extensively reviewed the history of studies focusing on the anatomy and physiology of the inner ear. The early studies of the inner ear anatomy are mainly based on observations and descriptions of dissected human corpses. In the 19th and 20th centuries, many comparative studies used dissection techniques, which enable the extraction of the inner ear endocast from the surrounding bone (Gray, 1907; Retzius, 1881, 1884). Hyrtl (1873) provided the most comprehensive visualization of the mammalian bony labyrinths. He modified his previously established procedure of “Corrosionsanatomie” (see methodology in Hyrtl, 1845), in which metal based or wax‐like fluids are filled in the cavity of the bony labyrinth before the bone is removed. In the early 1980s, the investigation of fragile fossil material with destructive methods was increasingly replaced by noninvasive computer tomography scanning (e.g., Conroy & Vannier, 1984; Hoffmann, Schultz, et al., 2014a), which provides a method to virtually reconstruct the internal anatomy of the bony labyrinth. As then, dozens of studies focus on aspects of phylogeny (e.g., Benoit et al., 2015; Lebrun, De León, Tafforeau, & Zollikofer, 2010; Maisey, 2001), physiology (e.g., Armstrong, Bloch, Houde, & Silcox, 2011; Coleman & Colbert, 2007; Kirk & Gosselin‐Ildari, 2009; Manoussaki et al., 2008), ontogeny (Billet, de Muizon, et al., 2015; Costeur, Mennecart, Müller, & Schulz, 2017; Ekdale, 2010; Mennecart & Costeur, 2016; Sánchez‐Villagra & Schmelzle, 2007), paleobiology (e.g., David et al., 2010; Neenan & Scheyer, 2012; Pfaff Nagel, et al., 2017; Spoor, Bajpai, Hussain, Kumar, & Thewissen, 2002) or functional morphology (e.g, Coutier, Hautier, Cornette, Amson, & Billet, 2017; Grohé, Tseng, Lebrun, Boistel, & Flynn, 2016; Pfaff, Czerny, Nagel, & Kriwet, 2017b; Pfaff, Martin, & Ruf, 2015; Ruf et al., 2016; Schellhorn, 2018a; Schutz, Jamniczky, Hallgrímsson, & Garland, 2014; Spoor et al., 2007) of the labyrinth organ in extant but also extinct taxa. Anatomical correlations between membranous and bony labyrinths seem underrepresented, and precise and detailed descriptions based on histological serial and thin sections are valuable (e.g., Maier, 2013; Maier & van den Heever, 2002; Schultz, Zeller, & Luo, 2017; Starck, 1995; Wever, 1978, 1985).

Even though Hyrtl (1873) doubted any correlation between the structure of the vestibular system and locomotion type, recent functional investigations of the inner ear morphology in a variety of vertebrates based on micro‐CT scanning clearly show that shape and locomotion are linked (e.g., Berlin, Kirk, & Rowe, 2013; Billet et al., 2012; Cox & Jeffrey, 2010; David et al., 2010; Malinzak, Kay, & Hullar, 2012; Pfaff et al., 2015; Pfaff, Czerny, et al., 2017b; Ryan et al., 2012; Spoor et al., 2007; Spoor, Wood, & Zonneveld, 1994; Spoor & Zonneveld, 1998). Even though, the results of these studies appear controversial between different orders (e.g., functional‐morphological distinction based on size of SCs vs. diameter of SCs), the diameter and arc size of the SCs are positively correlated among vertebrates and are not independent from one another (Muller, 1999). In addition to locomotion modes, also the head posture of the investigated specimens can be reconstructed (e.g., De Beer, 1947, Blanks, Curthoys, & Markham, 1972, Coutier et al., 2017, contradictory view: Spoor, 2003) and, therefore, feeding preferences in their lifestyle (Schellhorn, 2018a). Most of these studies assume that the anatomy of the vestibular system is quite conservative. However, in some mammalian groups (e.g., Xenarthrans) the anatomy of the bony labyrinth is highly divers (Billet et al., 2012). Besides functional and morphological adaptations, phylogenetic signals can be seen in the anatomy of the vestibular system (e.g., Billet, Hautier, & Lebrun, 2015; Mennecart & Costeur, 2016).

The shape and number of coils of the mammalian cochlea is known to have a strong phylogenetic signal (Ekdale, 2016a). Monotremes and early mammaliaforms show less than 360° (Luo, Ruf, & Martin, 2012; Luo, Ruf, Schultz, & Martin, 2011; Rowe, 1996; Ruf, Luo, & Martin, 2013; Ruf, Luo, Wible, & Martin, 2009). In monotremes, the cochlear apex containing the lagena is coiled and enlarged (Schultz et al., 2017), while gondwanatherian mammals show a short tapering cochlear canal with a slightly curved apex (Hoffmann, O'Connor, Kirk, Wible, & Krause, 2014). Therian mammals (marsupials and placentals) show at least one full turn but can have more than three turns (e.g., 3.25 in domestic cats [Schellhorn, 2018b], or 3.3 in whales [Ekdale, 2016b]). Furthermore, the shape of the cochlear is phylogenetically informative in whales (toothless: Ekdale, 2016b; toothed: Costeur et al., 2018).

The evolution of the amphibian inner ear on its way to amniotes is controversially discussed (Lombard, 1977; Romer & Price, 1940; Wever, 1974, 1976). The course of the connection between the periotic tube represented in amphibians as the periotic canal and in amniotes as the scala vestibuli, helicotrema and scala tympani, differs substantially, which questions the homology of these structures (Wever, 1976; reviewed by Lombard & Bolt, 1979) and, therefore, disagreement about the homology of the amphibian basilar papilla with the organ of corti exists. The papilla amphibiorum of anurans was shown not to be homologous to the basilar papilla of nonmammalian amniotes and the organ of Corti of mammals (de Burlet, 1934; but see Fleischer, 1973). However, it is commonly assumed that the amniotic basilar papilla is the positional homolog to the therian organ of Corti although it is histologically distinct (see Fritzsch et al., 2013). Additionally, it is postulated that during the mammalian evolution the sensory basilar papilla of the lagena was integrated into the apex of the organ of Corti at the apex of the cochlear duct. This evolutionary transformation presumably had occurred after the monotreme‐therian split, and within the theriimorph evolution (Schultz et al., 2017).

### 1.3 FUTURE PERSPECTIVES

Since the expanding availability of noninvasive microcomputed tomography anatomical investigations of the middle and inner ear region of extant and extinct vertebrates are of still growing interest to the scientific community addressing complex questions. For example, the evolution of the huge diversity of the membranous inner ear labyrinth morphology, the function of the vestibular system for detecting acceleration and deceleration of the head and related transmission of sensory signals, the evolution of the mammalian coiled cochlea for enhanced hearing capability, and many more. Recently conducted studies focus on phylogenetic signals to contribute to the tree of life and reveal ecological adaptations. The goal of the symposium held at the 11th ICVM was to exchange newly acquired insights, new technical approaches, and exchange of information addressing anatomical meaning of the bony structure of inner and middle ear related structures in the system of vertebrates.

## CONFLICT OF INTERESTS

There are no Conflict of interests.

## AUTHORS' CONTRIBUTIONS

All authors drafted the manuscript, and gave final approval for publication.
